# Naturally conceived heterotopic pregnancy: an atypical presentation rare case report and review of current literature

**DOI:** 10.1093/jscr/rjae373

**Published:** 2024-05-31

**Authors:** John Lugata, Baraka Shao, Nasra Batchu

**Affiliations:** Department of Obstetrics and Gynecology, Kilimanjaro Christian Medical Centre, PO Box 3010, Moshi, Tanzania; Faculty of Medicine, Kilimanjaro Christian Medical University College, PO Box 2240, Moshi, Tanzania; Department of Obstetrics and Gynecology, Kilimanjaro Christian Medical Centre, PO Box 3010, Moshi, Tanzania; Faculty of Medicine, Kilimanjaro Christian Medical University College, PO Box 2240, Moshi, Tanzania; Department of Obstetrics and Gynecology, Kilimanjaro Christian Medical Centre, PO Box 3010, Moshi, Tanzania; Faculty of Medicine, Kilimanjaro Christian Medical University College, PO Box 2240, Moshi, Tanzania

**Keywords:** heterotopic pregnancy, ectopic pregnancy, salpingectomy, cesarean section and laparotomy

## Abstract

Heterotopic pregnancy (HP) is the coexistence of living or dead intrauterine pregnancy, single or multiple, and extrauterine pregnancy located in the oviduct, ovary, uterine corner, cervix or peritoneal cavity. This condition is very rare (1:30 000 pregnancies). HP constitutes a rare obstetric condition. Its occurrence after natural conception is sparsely documented in the literature. Here in, we present a case of a 27-year-old primeparous women who presented at 18 weeks with features of ruptured ectopic pregnancy. Initial ultrasonographic imaging showed an intrauterine pregnancy corresponding to 18 weeks. It also revealed a floating fetus with significant collection of fluid in the pouch of Douglas, retroceacal recess and both hepatocellular recess. An emergency explorative laparotomy was done where right salpingectomy was performed. She was later followed up to term and delivered by elective cesarean section successfully. A brief narrative of the challenges in the management, clinical presentation and limitation in the management is highlighted in the present case report.

**Key message:** Heterotopic pregnancy can occur in natural conception irrespective of usage of ovulation induction. Routine early pregnancy ultrasound can promote early detection with prompt surgical intervention to mitigate its complications.

## Introduction

Heterotopic pregnancy (HP) refers to a dual coexistence of both extra-uterine and intrauterine pregnancy. It represents an extremely rare obstetric condition with distinct documented clinical presentations and complications. The documented occurrence rates is estimated to be 1 to 30 000 deliveries [1] with high occurrence (1 in 100) in women undergoing assisted reproductive technique ART [2]. Majority of the documented reports in the literature are ART-related cases [3–6] with natural conceived HP reports being virtually non-existent.

The presentation of ruptured HP constitutes a clinical challenge in diagnosis and optimal management. Its presentation in resource constrained setting poses additional intricacy in diagnosis, given the unavailability of high-resolution imaging and expertise to offer timely diagnosis and management.

The most common site for ectopic in HP is ampulary—similar to the isolated ectopic pregnancy. The clinical presentations are variable; early first trimester abdominal pain, per vaginal bleeding that occasionally results in abortion, which has been reported in the literature. A considerable proportion (60–70%) of the HP may proceed to term and deliver normally. Delay diagnosis may result in significant maternal and fetal morbidity that can be fatal if unattended timely.

We herein provide a detailed narration of the clinical presentation of an HP that presented with ruptured cornual pregnancy at a gestation of 18 weeks, which was managed surgically and later proceeded to full term and delivered successfully by cesarean section.

## Case presentation

A 27-year-old lady presented to our facility with the complaint of sudden onset of severe abdominal pain for a duration of 1 day. She described her pain to be sharp in nature along the suprapubic areas radiating to the peri-umbilical area accompanied with generalised body malaise with light headedness for the same duration. She also had a history of amenorrhoea for a duration of 9 weeks. There were no obvious relieving or aggravating factors. Her past obstetric history was of a previous vaginal delivery. She had a menarche at an age of 15 years, had a regular preconception menstrual flow of 28 days, with a 5-day duration. She is married and works as a petty trader without any history of smoking cigarette or drinking alcohol. She had no history of usage of ovarian stimulation medication nor any prior history of usage of contraceptives.

On examination, she was alert and severely pale afebrile (*T* = 37.2°C) with a bood pressure of 100/60 bpm, a pulse rate of 120 beats per minute and oxygen saturation of 99% in room air. Upon abdominal examination, the fundal height was 18/40 weeks with easily palpable fetal parts. She had rebound tenderness and muscle guarding. Sterile speculum examination revealed a closed porous cervix with a slight blood stain; other parameters were essentially unremarkable.

A series of investigations were done as part of the baseline initial work up. A complete blood count revealed a hemoglobin level of 12.3 g dl, a hematocrit level of 33 and a platelet count of 230/μl. Aspartate transaminases was 16.5 m mol^−1^ with creatinine of 60 μm mol^−1^, urea of 1.9 μm mol^−1^. An ultrasound revealed an intrauterine pregnancy corresponding to 18 weeks. It also revealed a floating fetus with significant collection of fluid in the pouch of Douglas, retroceacal recess and both hepatorenal recess.

**Figure 1:**
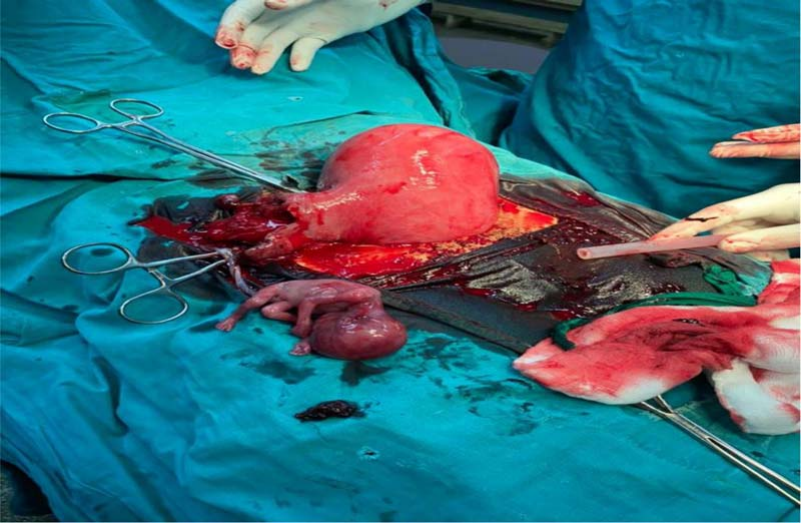
A ruptured right tubal pregnancy with an intrauterine pregnancy.

**Figure 2:**
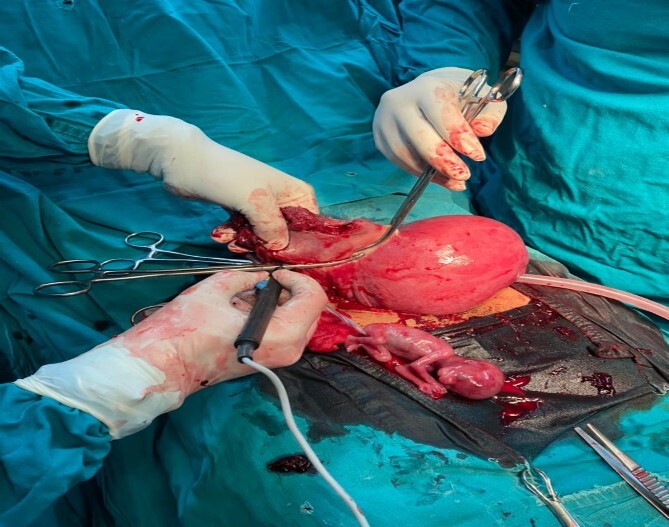
A resection of the right tubal ruptured ectopic pregnancy.

Given the presentation and radiological findings, a prompt surgical intervention was arranged. Two units of blood were prepared for surgery. She was then given a general anaesthesia, Intraoperatively, a pfannenstiel incision was made and encountered significant hemoperitoneum of 500 ml with clots. Rupture right cornual extended to the isthmus with a floating fetus (as shown in Fig. 1). A wedge resection of the right cornu-ishtmic portion of the tube was done (as shown in Fig. 2). Peritoneal lavage with warm normal saline ~1 le was also done. Post-operatively, she was kept on tocolytic agent-Nifedipine 20 mg once a day for a duration of 1 week.

Thereafter, she was kept in the ward for a duration of 5 days. She was hemodynamically stable and discharged 5 days after surgery and was counseled to return twice monthly for a duration of 2 months for a continual obstetric surveillance. All visits in the antenatal care depicting normal fetus with an appropriate growth pattern and a normal biophysical profile. She was then scheduled for an elective cesarean delivery after completing 38 weeks of gestation.

**Figure 3:**
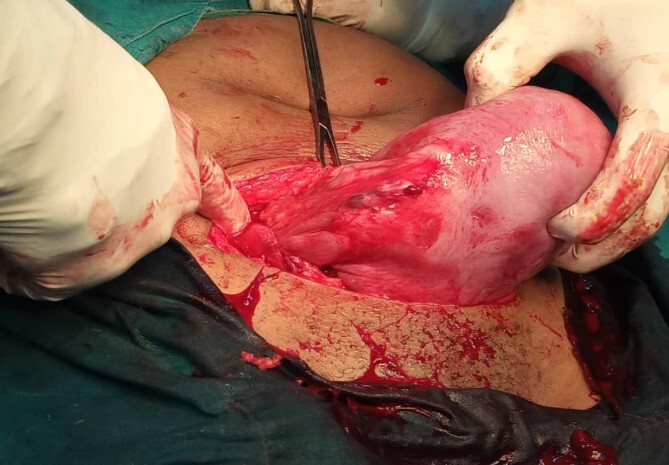
A healed right adnexa as it appears after cesarean section.

After 39 weeks, she was admitted and delivered by a cesarean section. A 3.2-kg female baby was extracted who had an apgar score of 9/10 in 1/5 minutes, respectively. The previous cornual scar had healed (as shown in Fig. 3).The patient was kept on post-operative care including analgesics, antibiotics, intravenous fluid infusions, hematinics, and she had smooth recovery and was discharged home in a stable condition on day 5 after surgery to return after 2 weeks for a follow-up.

## Discussion

To date, there is a consensus on the mechanism for ectopic implantation. Several postulates have been implicated in the literature including tubal anatomical distortion, impaired tubal motility function with a possibility of transperitoneal migration of the embryo. Anatomical distortion of the tubes is however a widely accepted mechanism. This usually results from pelvic inflammatory disease, diverticulosis of the fallopian tube (isthmic nodosa), with fibrosis of the fallopian tube following infective process or previous tubal surgery. Pathological evidence of fibrotic changes in the uterine tubes among tubal ectopic cases suggests anatomical occlusive mechanism, but the absence of fibrotic tubal features suggests other mechanism sfor ectopic formation. In our case, it remains intuitively challenging to postulate for an occlusive tubal features on the site of ectopic since she had intrauterine pregnancy. Conversely, a supra-ovulation might occur, resulting in intrauterine pregnancy via the unaffected tube. Although we had no clinical evidence of supra-ovulation following the usage of ovulation inducing agents, an alternative mechanism would be a transperitoneal migration of the embryo from the affected side to result in intrauterine pregnancy via a contralateral tube [7]. Most of the evidence in the literature for the transperitoneal migration of the embryo is evidence of corpus luteum in a non-communication tube in unicornuate uterus [8–10].

Majority of HP are reported to be diagnosed at a gest age of 5 and 8 weeks of pregnancy [1, 11]. In contrast, our case was diagnosed at a gest age of 18 weeks; partly, a reflection of challenges in early ultrasound imaging in our routine antenatal care. This has been reported elsewhere [12]. The clinical presentation of HP is variable and non-specific. Early in first trimester, HP is usually asymptomatic. Abdominal pain is reported to be the second most common presentation, in either subacute or acute state, provided it is ruptured, twisted or intact [13]. Vaginal bleeding occurs in approximately 24% of all cases of HP [14]. In our case, since the patient presented lately with ruptured ectopic, she presented with abdominal pain followed with vaginal bleeding.

Diagnosis of HP in early pregnancy presents a peculiar clinical dilemma, given its rarity. Most of the HP pregnancies are usually diagnosed clinically following ruptured ectopic [15]. Routinely used investigation including transvaginal ultrasound, may detect adnexae masses, but in view of coexisting intrauterine pregnancy, clinicians are more inclined to have an impression of other adnexae masses including theca letual cyst, hemorrhagic or other functional ovarian cyst rather than an ectopic mass. Standard complimentary tests including serial serum β-hcg levels may not necessarily provide added information since high levels form intrauterine pregnancy, which interferes with the usual diagnostic patterns of ectopic pregnancy.

Management of HP, once diagnosed, requires an expeditious surgical management of laparotomy and salpingectomy or wedge resection if it is cornual. In our case, laparotomy was undertaken and resection of the cornual section was conducted. She was later followed up in our antenatal clinic and planned for elective c/section at 39 completed weeks.

Several limitations are inherent in this current case report. First, there were no ultrasound images available from our radiology unit to share in this report. This has prohibited our ability to clearly discuss radiological challenges encountered in diagnosing this case. Secondly, we didn’t have the histology report of the uterine tube on the affected side, which prevented us to hypothesise potential mechanism for this HP.

## Conclusion

HP, following natural conceptions, is a rare clinical presentation. Detection of HP in advanced gestation seen in this particular case underscores the importance of early antenatal scan. Additionally, HP is an important differential for intrauterine pregnancy coexisting with adnexal masses. Once the diagnosis is made, prompt surgical management should be provided and close follow up of the women to term.
